# Selenium Antagonizes Cadmium-Induced Inflammation and Oxidative Stress via Suppressing the Interplay between NLRP3 Inflammasome and HMGB1/NF-κB Pathway in Duck Hepatocytes

**DOI:** 10.3390/ijms23116252

**Published:** 2022-06-02

**Authors:** Zhanyou Cao, Fan Yang, Yiqun Lin, Jiyi Shan, Huabin Cao, Caiying Zhang, Yu Zhuang, Chenghong Xing, Guoliang Hu

**Affiliations:** College of Animal Science and Technology, Jiangxi Agricultural University, No. 1101 Zhimin Avenue, Economic and Technological Development District, Nanchang 330045, China; czy704819938@163.com (Z.C.); Yfan@jxau.edu.cn (F.Y.); lyq022012@163.com (Y.L.); ShanJYCrow@163.com (J.S.); chbin20020804@163.com (H.C.); zhangcaiying0916@163.com (C.Z.); zhuangyu201212@163.com (Y.Z.)

**Keywords:** cadmium, selenium, HMGB1, inflammation, oxidative stress, hepatocytes

## Abstract

Cadmium (Cd) is a toxic heavy metal that can accumulate in the liver of animals, damaging liver function. Inflammation and oxidative stress are considered primary causes of Cd-induced liver damage. Selenium (Se) is an antioxidant and can resist the detrimental impacts of Cd on the liver. To elucidate the antagonism of Se on Cd against hepatocyte injury and its mechanism, duck embryo hepatocytes were treated with Cd (4 μM) and/or Se (0.4 μM) for 24 h. Then, the hepatocyte viability, oxidative stress and inflammatory status were assessed. The findings manifested that the accumulation of reactive oxygen species (ROS) and the levels of pro-inflammatory factors were elevated in the Cd group. Simultaneously, immunofluorescence staining revealed that the interaction between NOD-like receptor pyran domain containing 3 (NLRP3) and apoptosis-associated speck-like protein (ASC) was enhanced, the movement of high-mobility group box 1 (HMGB1) from nucleus to cytoplasm was increased and the inflammatory response was further amplified. Nevertheless, the addition of Se relieved the above-mentioned effects, thereby alleviating cellular oxidative stress and inflammation. Collectively, the results suggested that Se could mitigate Cd-stimulated oxidative stress and inflammation in hepatocytes, which might be correlated with the NLRP3 inflammasome and HMGB1/nuclear factor-κB (NF-κB) signaling pathway.

## 1. Introduction

Cadmium (Cd) is a biologically non-essential trace element broadly present in water, the atmosphere and soil [1]. Human activities such as mining and industrial manufacturing are the dominant origins of Cd contamination [2]. In contaminated areas, estimated concentrations of cadmium in sewage range from 0.21 to 11.8 mg/kg (EPA, 2009a), well above concentrations (<0.005 mg/L) in normal freshwater [3]. The concentration of Cd in soil water was reported to be 27.8 μg/L in northern Germany and as high as 6000 μg/L in groundwater around US waste sites [4]. Ducks are one of the major waterfowl. In certain zones and seasons, they may be exposed to Cd-contaminated water, particularly when they gather intensively in relatively sealed shallow waters. They are also more susceptible to Cd in water. Additionally, as a major source of protein for human beings, Cd in ducks can enter the body through the food chain and accumulate, causing harm. Previous studies proved that Cd could lead to chronic kidney disease and bone injury [5]. After ingestion of contaminated water and food by a human or animal, Cd primarily aggregates in the form of Cd-metallothionein complex in the liver and kidneys, and its turnover rate is excessively slow, which can disrupt the stability of essential metals such as zinc (Zn) and cuprum (Cu), reduce the capacity of cells to purge reactive oxygen species (ROS), cause oxidative stress and inflammation, and ultimately undermine organ function [6,7].

NOD-like receptor protein 3 inflammasome (NLRP3 inflammasome) is a multi-protein complex. Its excitation touches on numerous pathological processes and plays a pivotal role in the inflammatory phase, leading to cell death in the mode of inflammation [8,9]. The binding of NLRP3 pro-cysteinyl aspartate specific proteinase (caspase)-1 makes apoptosis-associated speck-like protein (ASC) serve as a bridge to excite NLRP3 inflammasome, thereby autocatalyzing caspase-1 and contributing to the elevated emission of pro-inflammatory cytokines [10]. According to research, the excitation of NLRP3 inflammasome played a crucial function in nonspecific inflammation of inflammatory bowel disease (IBD) [11]. Ka et al. testified that suppressing the activation of NLRP3 inflammasome and intensifying the stimulation of Nrf2 could mitigate the course evolution of accelerated severe lupus nephritis (ASLN) [12]. Furthermore, the activation of NLRP3 inflammasome concerns multiple cellular signaling mechanisms, comprising ROS accumulation, potassium efflux, and lysosomal trauma, among which ROS is the firsthand stimulator of NLRP3 inflammasome excitation [13,14]. Previously, it was pointed that the cumulative ROS excited NLRP3 inflammasome and caused an inflammatory response in hepatocytes [15,16]. In addition, pro-inflammatory factors, such as interleukin (IL)-1β and IL-18, exert a crucial role in anti-infection defense. However, the excessive release of these cytokines and the accumulation of ROS can lead to uncontrolled systemic inflammation and stimulate the activation of other inflammation-related pathways, such as the high-mobility group box 1 (HMGB1)/nuclear factor-κB (NF-κB) pathway [17].

One of the main roles of HMGB1 is that, as a damage-related molecular pattern molecule (DAMP), it migrates from the nucleus to the cytoplasm and discharges to the outside of the cells, and then binds to interrelated receptors (such as Toll-like receptors (TLRs) and advanced glycation end products (RAGE)) to exert pro-inflammatory effects [18]. TLR4 is a well-studied member of the TLRs family, and it is also one of the principal acceptors for discriminating and linking extracellular HMGB1. After binding to HMGB1, TLR4 dimerizes and recruits myeloid differentiation primary response protein 88 (MYD88) to reversibly covalently modify NF-κB, triggering a cascade of reactions [19,20]. Liu et al. proved that exposure to amorphous SiO2 NP could promote the release of HMGB1 from the nucleus to the cytoplasm, thus activating the NF-κB signaling pathway, leading to inflammatory injury of human umbilical vein endothelial cells (HUVEC) [21]. Recent studies have shown that Cd can produce a series of adverse effects, including increasing autophagy levels and promoting apoptosis, endoplasmic reticulum stress and pyroptosis in vivo or in vitro experimental models [22,23,24,25]. Furthermore, these undesirable reactions are accompanied by the addition of inflammatory cytokines and the enhancement of inflammatory levels. The pro-inflammatory factors (such as IL-1β and IL-18) are released in the early stage of inflammation, while HMGB1 release is delayed and persistent [26]. When excessive ROS and pro-inflammatory factors are produced in cells, the HMGB1/NF-κB pathway will be activated to produce more pro-inflammatory factors, forming a positive feedback loop, and causing an uncontrollable inflammatory reaction. Previous defined extracellular HMGB1 is crucial to the late mediator of experimental sepsis and could be targeted for treatment within a wider therapeutic window [27]. Thus, the inhibition of oxidative stress and inflammatory response in hepatocytes may be a momentous entry point to alleviate Cd-induced hepatocyte lesions.

Selenium (Se) is a requisite trace element for cells, mainly in the form of inorganic Se (such as selenate and selenite) and organic Se (such as Se amino acids, Se polysaccharides, Se-containing proteins) in food [28]. Recent reports have shown that different forms of Se, such as selenite, selenomethionine, Se from lentils, or nano-Se, can reduce Cd-mediated kidney, liver, brain and cardiotoxicity in animal models and cell culture studies [29]. Se also has an antioxidant effect, which can restrain the formation of ROS by increasing the activity of glutathione peroxidase (GSH-Px) [30]. The liver is the central metabolic organ of Se in animals, maintaining the balance of Se [31]. A study showed that Se could protect chicken liver damage induced by Cd through inhibiting oxidative stress [32]. In addition, Qu et al. reported that Se inhibits liver tissue inflammation induced by lipopolysaccharide (LPS) by restraining the TLR4/NF-κB/NLRP3 signaling pathway [33]. Previous studies found that Cd could cause oxidative stress and inflammatory re-action in rats [23,34]. Additionally, Zhang et al. discovered that Cd reduced the activities of GSH-Px, SOD and T-AOC, and increased the content of MDA in rabbit liver, which caused oxidative stress, and the addition of Se alleviated the oxidative stress caused by Cd [35]. At present, the methods used for the treatment of Cd poisoning mainly include the use of chelating compounds such as calcium disodium versenate and nutritional interventions with Se and vitamins [36,37]. Se has a strong liver protection effect, but so far, few studies have reported the effects of Cd and Se on poultry liver, and the exact antagonistic mechanism between Se and Cd and the intervention mechanism of inflammatory reaction are still uncertain. This experiment enriches the research of Cd and Se in liver. Moreover, in comparison with other mammals, most waterfowl generally forage in rice fields and rivers, and are more vulnerable to the exterior environment and diseases. Waterfowl, particularly ducks, are one of the largest breeding birds in the world, and the main source of animal protein for humans, especially in China [38]. Therefore, duck hepatocytes exposed to Cd and Se were taken as the research subjects in this experiment, and ROS production, the related indicators of oxidative stress and inflammation in hepatocytes were detected. We studied the relationship between the alleviating effect of Se on oxidative stress and inflammation induced by Cd, NLRP3 inflammasome and the HMGB1/NF-kB signaling pathway. The purpose of this study is to explore the hepatotoxicity of Cd and the antagonism of Se, and to provide a theoretical basis for the study of liver toxicology and environmental protection of Cd.

## 2. Results

### 2.1. Selection of Cd and Se Dosage

**Figure 1:**
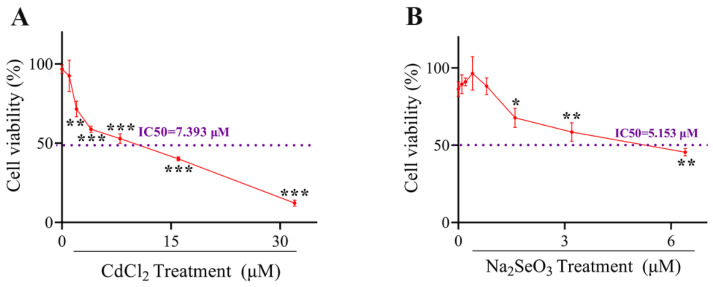
The cells viabilities under Cadmium (Cd) or Selenium (Se) treatment. (**A**) The cells viability curve of hepatocytes exposed to CdCl_2_ at different concentrations (0, 1, 2, 4, 8, 16 and 32 μM) for 24 h. (**B**) The cells viability curve of hepatocytes exposed to Na_2_SeO_3_ at different concentrations (0, 1, 2, 4, 8, 16 and 32 μM) for 24 h. The data are expressed as mean ± SD. “*” indicates a significant difference compared with control group (* *p* < 0.05, ** *p* < 0.01 and *** *p* < 0.001).

Hepatocytes were grown on a 96-well plate at a proper cell density and the cells were exposed to different concentration gradients of CdCl_2_ (0, 1, 2, 4, 8, 16 and 32 μM) and Na_2_SeO_3_ (0, 0.1, 0.2, 0.4, 0.8, 1.6, 3.2 and 6.4 μM) for 24 h. The viability of the cells of each group was shown in Figure 1A,B. When the Cd concentration was higher than 0.1 μM, Cd induced a significant decrease (*p* < 0.01 or *p* < 0.001) in the viability of the cells in a dose-dependent manner. The half-maximal inhibitory concentration (IC50) of CdCl_2_ used on hepatocytes is 7.393 μM. Furthermore, when cells were exposed to different doses of Na_2_SeO_3_, the cell viability ascended in a dose-dependent manner until 0.4 μM, then the viability of the cells decreased in a dose-dependent manner and decreased significantly from 1.6 μM (*p* < 0.05 or *p* < 0.01). Therefore, 4 µM Cd and 0.4 µM Se were selected in further experiments.

### 2.2. Se Alleviates Cd-Induced Hepatocytes Injury

**Figure 2:**
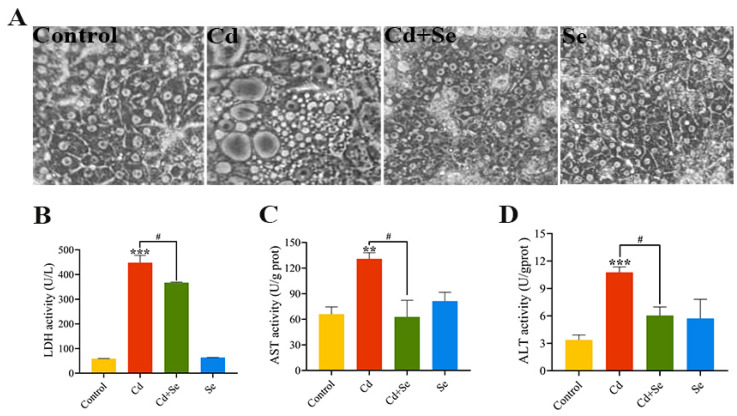
Se attenuates Cd-induced hepatocytes trauma. (**A**) The status of hepatocytes (400×); (**B**) lactate dehydrogenase (LDH) activity; (**C**) Aspartate aminotransferase (AST) activity; (**D**) Alanine aminotransferase (ALT) activity. The data are expressed as mean ± SD. “Asterisk” indicates a significant difference compared with control group (** *p* < 0.01 and *** *p* < 0.001). “^#^” indicates a significant difference between Cd group (^#^ *p* < 0.05).

The morphological observation of duck hepatocytes treated with Cd and/or Se for 24 h is shown in Figure 2A. The cell structure of the control group and Se group are intact with clear boundaries. By contrast, in the Cd group, the intercellular space is widened, cells have shrunken, and density has decreased, and many vacuoles have emerged, revealing that Cd has evident cytotoxicity to duck hepatocytes. However, in the Cd + Se group, the phenomena, as mentioned earlier, is alleviated.

### 2.3. Se Suppresses Cd-Stimulated Increases in ALT, AST and LDH Release from Hepatocytes

After the cells were processed with Cd and/or Se for 24 h, the activities of LDH, ALT and AST in the cell supernatant were evaluated, and the outcomes are illustrated in Figure 2B–D. Compared with the control group, the LDH activity in the Cd group was remarkably increased (*p* < 0.001). However, the LDH activity in the Cd + Se group was considerably depressed (*p* < 0.05) compared to the Cd group. Similarly, the activities of aminotransferase ALT and AST were evidently elevated (*p* < 0.01 or *p* < 0.001) in the Cd group compared with the control group. Additionally, the activities of ALT and AST in the Cd + Se group were greatly reduced (*p* < 0.05) in comparison with the Cd group.

### 2.4. Se Relieves Cd-Induced Oxidative Stress and Inflammation of Hepatocyte

**Figure 3:**
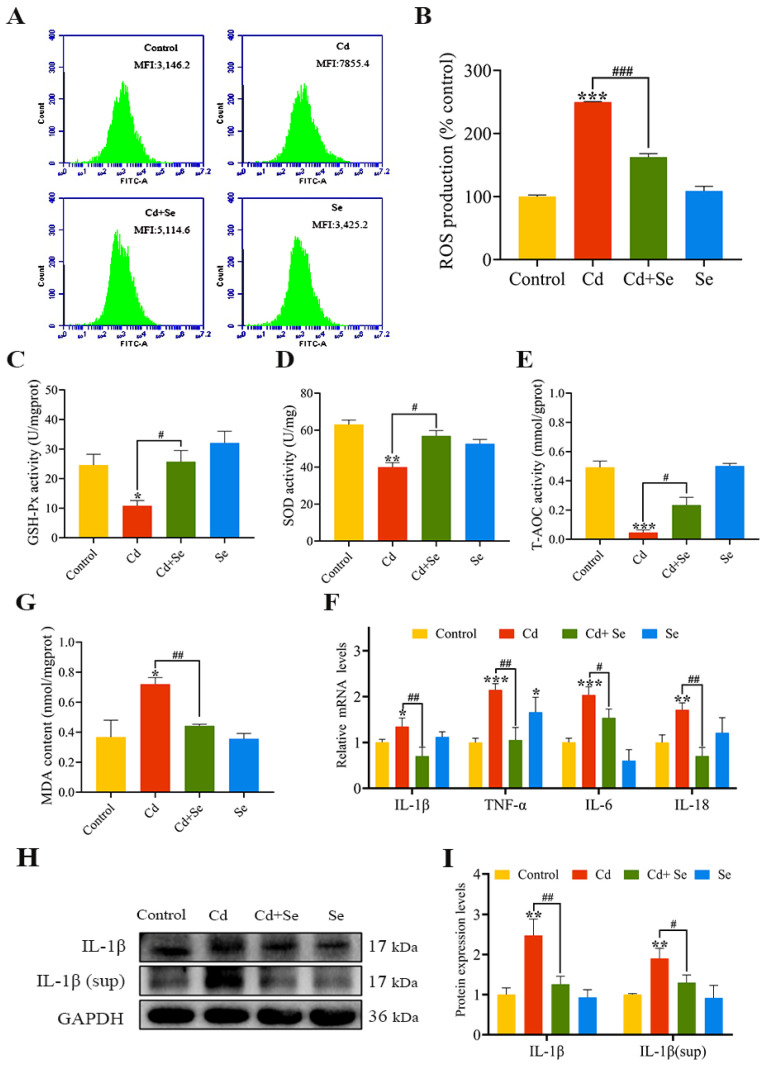
Se protects against hepatocellular oxidative stress and inflammation caused by Cd. (**A**,**B**) Reactive oxygen species (ROS) level; (**C**) Glutathione peroxidase (GSH-Px) activity; (**D**) Superoxide dismutase (SOD) activity; (**E**) Total antioxidant capacity (T-AOC) activity; (**F**) Malondialdehyde (MDA) content; (**G**) The mRNA levels of IL-1β, tumor necrosis factor (TNF)-α, IL-6 and IL-18; (**H**) Protein band graph; (**I**) The protein level of IL-1β. (sup represents released extracellular protein of cells in culture supernatant). The data are expressed as mean ± SD. “*” indicates a significant difference compared with control group (* *p* < 0.05, ** *p* < 0.01 and *** *p* < 0.001). “^#^” indicates a significant difference between Cd group (^#^ *p* < 0.05, ^##^ *p* < 0.01 and ^###^ *p* < 0.001).

The ROS fluorescence intensity results are shown in Figure 3A,B. The intracellular ROS levels in the Cd group were considerably higher than those in the control group (*p* < 0.001). Furthermore, in comparison with the Cd group, the levels of ROS in the Cd + Se group were significantly reduced (*p* < 0.001). The activities of GSH-Px, SOD and T-AOC in the Cd group were markedly lower than those in the control group (*p* < 0.05 or *p* < 0.01 or *p* < 0.001), whereas the content of MDA was obviously higher (*p* < 0.05). Nevertheless, in the Cd + Se group, the activities of GSH-Px, SOD and T-AOC were dramatically increased (*p* < 0.05), and the content of MDA was tremendously declined (*p* < 0.01) compared with the Cd group (Figure 3C–F).

Moreover, we measured the mRNA levels of inflammation-relevant factors (TNF-α, IL-1β, IL-18 and IL-6), as depicted in Figure 3G,H. Compared with the control group, the mRNA levels of these four factors in the Cd group were notably enhanced (*p* < 0.05 or *p* < 0.01 or *p* < 0.001). However, this increment was clearly restrained in the Cd + Se group (*p* < 0.05 or *p* < 0.01) (Figure 3G). The protein levels of IL-1β in hepatocytes and cell supernatant culture medium are are shown in Figure 3H,I. Compared with the control group, the protein levels of IL-1β in the hepatocytes and cell supernatant in the Cd group were increased (*p* < 0.001), but the level of IL-1β protein in the Cd + Se group was evidently lower than that in the Cd group (*p* < 0.05 or *p* < 0.01).

### 2.5. Se Represses Cd-Induced Activation of NLRP3 Inflammasomes

**Figure 4:**
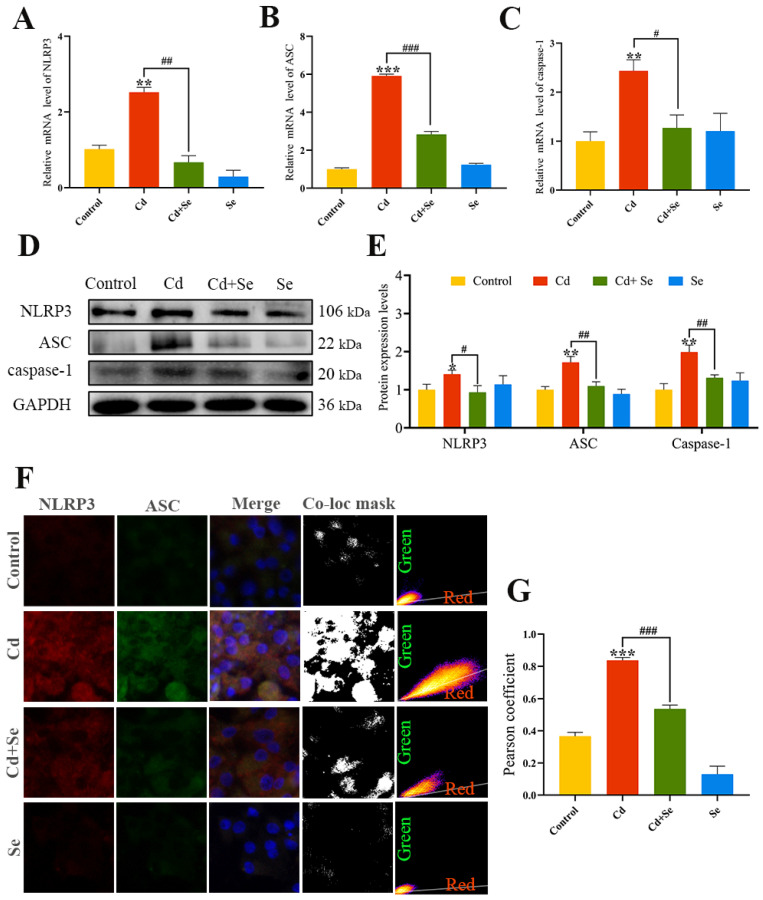
Se suppresses the activation of NOD-like receptor pyran domain containing 3 (NLRP3) inflammasome induced by Cd. (**A**–**C**) The mRNA expression levels of NLRP3, apoptosis-associated speck-like protein (ASC) and cysteine aspartic acid protease 1 (caspase-1); (**D**) Protein band graph; (**E**) The protein level of NLRP3, ASC and Caspase-1; (**F**,**G**) Co-localization analysis of NLRP3 and ASC. Green: NLRP3; Red: ASC; The mask of co-localization object (Co-loc mask). The data are expressed as mean ± SD. “*” indicates a significant difference compared with control group (* *p* < 0.05, ** *p* < 0.01 and *** *p* < 0.001). “^#^” indicates a significant difference between Cd group (^#^ *p* < 0.05, ^##^ *p* < 0.01 and ^###^ *p* < 0.001).

RT-qPCR and Western blotting results (Figure 4A–E) verified that the mRNA and protein levels of NLRP3, ASC and caspase-1 were remarkably increased in the Cd group compared with the control group (*p* < 0.05 or *p* < 0.01 or *p* < 0.001). In contrast, that in the Cd + Se group were markedly reduced in comparison with the Cd group (*p* < 0.05 or *p* < 0.01 or *p* < 0.001).

The results of the co-localization analysis of NLRP3 and ASC are shown in Figure 4F,G. Compared with the control group, the fluorescence signal of the interaction between NLRP 3 and ASC in the Cd group was obviously strengthened, and the Pearson coefficient of NLRP3 and ASC was increased noticeably (*p* < 0.001), making it closer to 1. The fluorescence intensity of Cd- and Se-treated hepatocytes at the NLRP3 and ASC intersection was distinctly reduced, and the Pearson coefficient was significantly lower compared to Cd treatment alone (*p* < 0.001).

### 2.6. Se Curbs Cd-Caused Activation of HMGB1/NF-κB Signaling Pathway in Hepatocytes

**Figure 5:**
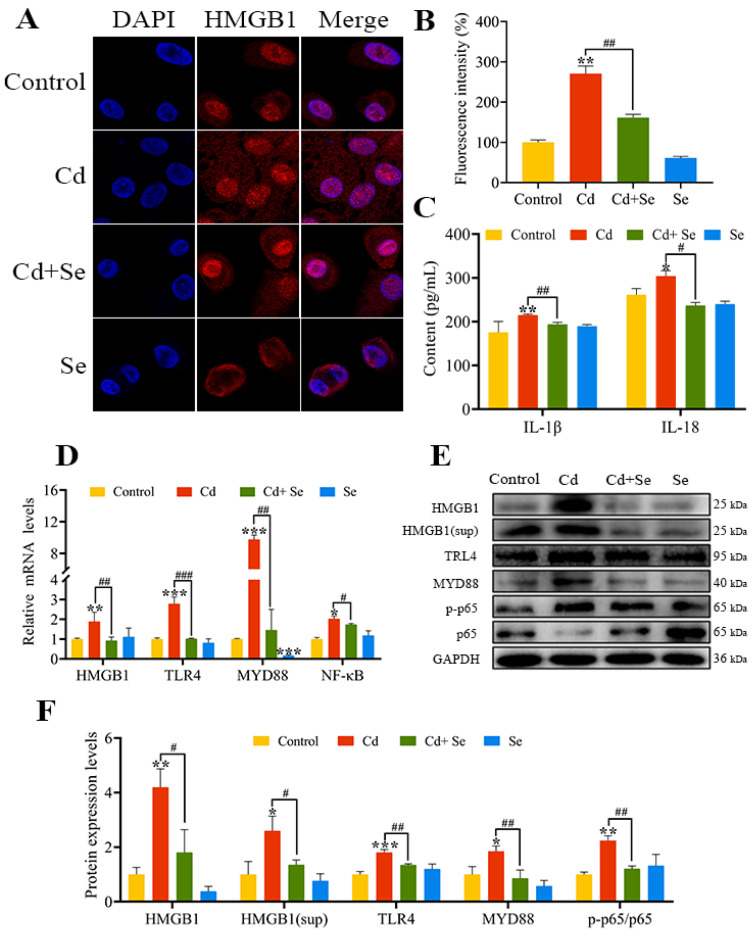
Se relieves the activation of high-mobility group box 1 (HMGB1)/nuclear factor-κB (NF-κB) signaling pathway induced by Cd. (**A**,**B**) HMGB1 immunofluorescence analysis. Blue: nucleus; Red: HMGB1; (**C**) The release of IL-18 and IL-1β in the culture medium; (**D**) The mRNA levels of HMGB1, myeloid differentiation primary response protein 88 (MYD88), Toll-like receptor (TLR) 4 and NF-κB; (**E**) Protein band graph; (**F**) The protein levels of HMGB1, MYD88, TLR4, p65 and p-p65. The data are expressed as mean ± SD. “*” indicates a significant difference compared with control group (* *p* < 0.05, ** *p* < 0.01 and *** *p* < 0.001). “^#^” indicates a significant difference between Cd group (^#^ *p* < 0.05 and ^##^ *p* < 0.01, ^###^ *p* < 0.001).

The HMGB1 immunofluorescence staining of hepatocytes is presented in Figure 5A. The fluorescence intensity in the Cd group was significantly stronger than that in the control group (*p* < 0.01). However, the fluorescence intensity in the Cd + Se group was apparently diminished compared with the Cd group (*p* < 0.01) (Figure 5B). The ELISA results are depicted in Figure 5C. After Cd exposure for 24 h, the concentrations of IL-1β and IL-18 in the cell supernatant were markedly increased in contrast to the control group (*p* < 0.05 or *p* < 0.01). Nevertheless, compared with the Cd group, their concentrations in Cd + Se were evidently reduced (*p* < 0.05 or *p* < 0.01).

In addition, the mRNA levels of HMGB1, MYD88, TLR4 and NF-κB in the Cd group were markedly increased in contrast to the control group (*p* < 0.05 or *p* < 0.01 or *p* < 0.001). The mRNA levels of HMGB1, MYD88, TLR4 and NF-κB in the Cd + Se group were noticeably lower than that in the Cd group (*p* < 0.05 or *p* < 0.01 or *p* < 0.001) (Figure 5D).

Consistently, in comparison with the control group, the protein levels of HMGB1, MYD88, TLR4 and p-p65 were notably upregulated in the Cd group (*p* < 0.05 or *p* < 0.01 or *p* < 0.001). Furthermore, the protein level of HMGB1, MYD88, TLR4 and p-p65 in the Cd + Se group was remarkably downregulated compared to the Cd group (*p* < 0.05 or *p* < 0.01) (Figure 5E,F).

### 2.7. HMGB1 Exerts a Vital Role in Se Restraining Cd-Induced Oxidative Stress and Inflammation of Hepatocytes

**Figure 6:**
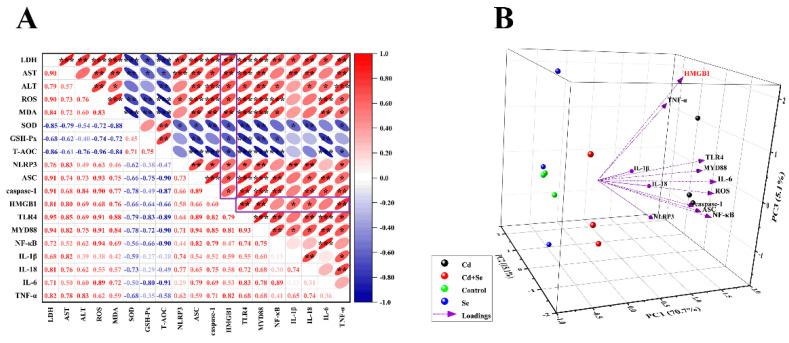
HMGB1 plays a vital role in Cd-induced hepatocyte damage. (**A**) Correlation analysis between factors related to hepatocyte damage. The color gradient indicates the correlation coefficient, and red is positive correlation, blue is negative correlation. (**B**) Ordination diagram of the principal component analysis (PCA) of HMGB1/TLR4/NF-κB signaling pathway related factors in hepatocytes induced by Cd or/and Se. The X, Y and Z coordinate axis are three principal components (PC1, PC2 and PC3), respectively, and the percentages in brackets are the percentage of explained variance. “*” indicates a significant correlation (* *p* < 0.05, ** *p* < 0.01 and *** *p* < 0.001).

Ultimately, we conducted a correlation analysis for all measured indices (Figure 6A). The outcomes indicated that the mRNA level of HMGB1 was positively correlated with the levels of hepatocyte damage-related factors (LDH, ALT, AST, ROS, MDA, NLRP3, ASC and caspase-1) (Pearson coefficient > 0.66, *p* < 0.05). However, the mRNA level of HMGB1 was negatively correlated with the activities of antioxidant enzymes (GSH-Px, SOD and T-AOC) (Pearson coefficient < −0.64, *p* < 0.05) (inside the box). In addition, NLRP3 inflammasome-related factors (NLRP3, ASC, and caspase-1) positively correlated with HMGB1/NF-κB-related factors (HMGB1, MYD88, TLR4 and NF-κB) (Pearson coefficient > 0.46, *p* < 0.05). Based on these results, principal component analysis (PCA) was performed on the factors interrelated to oxidative stress and inflammation of hepatocytes in each group. Three comprehensive variables (PC1 = 70.7%, PC2 = 15.1% and PC3 = 5.1%) representing the original variable information to the maximum were abstracted, and the three-dimensional space was obtained, as represented in Figure 6B. The results showed that the factors associated with NLRP3 inflammasomes (NLRP3, ASC, and caspase-1) and the the HMGB1/NF-κB pathway (HMGB1, MYD88, TLR4 and NF-κB) were numerically comparatively large in PC1. However, the values of HMGB1 in PC1, PC2 and PC3 were all large correspondingly, which proved that HMGB1 was a key factor affecting Cd-induced oxidative stress and inflammation in hepatocytes.

## 3. Discussion

Oxidative stress and inflammation are thought to be the groundwork of liver disease caused by factors including heavy metal accumulation, viral inflammation, alcohol, drugs, and other aspects [39]. Consistently, the current study revealed that Cd could cause oxidative stress and inflammatory damage in hepatocytes. For a long time, Se has been celebrated for its capacity to combat oxidative stress and the deleterious impacts of many toxic metals. Studies have confirmed that increasing Se ingestion might forestall the assimilation and toxicities of Cd [40,41]. Nonetheless, there are a few reports on the effects of Se on alleviating hepatic injury caused by Cd in which its mechanism is also uncertain. Consequently, in this study, the exposure model of duck primary liver cells in vitro was adopted to explore the toxic effects of selenium and cadmium on duck liver, focusing on the relationship between selenium, cadmium, oxidative stress, and inflammation-related NLRP3 inflammasome and the HMGB1/NF-κB pathway. Studies show that Cd enhanced ROS production, up-regulated antioxidant indices, activated NLRP3 inflammatory corpuscles and the HMGB1/NF-κB signaling pathway, and expanded the inflammation of hepatocytes. In addition, Se could alleviate Cd-induced inflammation and oxidative stress in duck hepatocytes.

Studies have shown that Cd is hepatotoxic and can cause hepatocyte damage [35,42]. When hepatocytes are damaged, the permeability of the cell membrane increases and the release of LDH and transaminases (ALT and AST) rises, which is an essential indicator for evaluating liver damage [43]. In the present study, the levels of ALT, AST and LDH after Cd treatment were markedly increased, and the vacuolation, atrophy and shedding of the cultured hepatocytes were multiplied, which demonstrated that Cd had a marked poisonous impact on hepatocytes. Furthermore, Cd could destabilize the oxidation and antioxidant balance of cells and organs [44]. There is only one oxidation state of Cd that will not directly generate free radicals but will affect the activity of antioxidant enzymes so that superoxide anion, hydroxyl radicals, etc. cannot be purged without delay to indirectly stimulate ROS buildup [45]. Excessive ROS have cytotoxicity effects on cells, disrupting the integrity of cell membranes and creating impairment [46]. Oxidative stress can be directly evaluated by measuring ROS, and ROS can affect the activities of related antioxidant enzymes (GSH-Px, SOD, MDA and T-AOC). GSH-Px is an indispensable peroxidase that extensively distributes in the organism, which can catalyze the conversion of GSH to GSSG and convert noxious peroxides to non-hazardous hydroxyl compounds. SOD can catalyze the disproportionation of superoxide anion free radicals to generate oxygen and hydrogen peroxide, thereby scavenging free radicals, which play a crucial role in the balance of oxidation and antioxidants in the body. MDA, as an important parameter reflecting the antioxidant capacity of the body, can not only reflect the rate and intensity of lipid peroxidation, but also indirectly reflect the degree of tissue peroxidation. Additionally, T-AOC determines total levels of antioxidant molecules, antioxidants and enzymes [47,48,49]. Se is a crucial component of many enzymes and antioxidants. When combined with proteins, it can exert a wide range of multi-effect roles, such as anti-oxidation and anti-inflammation [50,51]. According to our results, Cd caused the accumulation of ROS, decreased the levels of GSH-Px, SOD and T-AOC, and elevated the content of MDA, and Se could reverse the above changes, which proved that Cd caused oxidative stress in hepatocytes, and Se could antagonize oxidative stress caused by Cd.

Oxidative stress factors such as ROS are closely related to inflammation. Inflammation is the defensive response of the body to stimulation. The immune response of human and animal bodies to environmental factors, stress or injury signals depends on pattern recognition receptors (PRRs), which are closely coordinated by extracellular stimulation and intracellular receptors to produce adaptive changes [52]. NOD-like receptors (NLRs), one of the PRRs, are located in the cytoplasm and participate in the innate immunity of the organism [53]. NLRP3 inflammasome is a protein complex formed with the participation of NLRP3, ASC and Caspase-1, located intracellularly. Additionally, under normal conditions of the organism, their activation can release appropriate amounts of lL-1β and L-18, which participate in inflammatory immune response and maintain cellular homeostasis. However, the excessive activation of NLRP3 inflammasome can further the inflammatory response and cause repeated tissue damage [54]. The secretion of mature forms of IL-1β and IL-18 is the result of activation of NLRP3 inflammasome. Wu et al. verified that ROS triggered NLRP3 inflammasome, leading to a series of inflammatory reactions such as increased secretion levels of IL-1β and IL-18 [55]. Meanwhile, Hoffmann et al. have shown that Se strongly affects inflammation and enhances the immune response [56]. Additionally, Se is helpful in treating testicular injury induced by NLRP3 inflammasome [57]. Therefore, we speculated that Se could also alleviate duck hepatocyte inflammation caused by Cd exposure. Then, we detected the related indices and found that Cd could dramatically up-regulate the expression of IL-18, IL-1β, NLRP3, ASC and caspase-1, and the fluorescence signal of the interaction between NLRP3 and ASC was clearly enhanced, which meant that Cd activated NLRP3 inflammasome. Based on the concentration of Cd in the environment, Cao et al. fed ducks with a basal diet containing 4 mg/kg Cd for 16 weeks and the results suggested the expression of NLRP3, ASC, IL-18 and IL-1β was significantly increased, resulting in liver inflammation, which is consistent with our results [42]. In addition, the co-treatment of Se and Cd could greatly ameliorate the above changes and lower the expression level of NLRP3 inflammasome and other related inflammatory factors. According to previous reports, NLRP3 could promote the release of HMGB1 through inflammasome [58]. HMGB1 is an essential inflammatory factor, which could be released by damaged cells [59]. The migration and liberation of HMGB1 will activate the downstream signal pathway, further accelerating the release of related pro-inflammatory factors, and lead to uncontrollable inflammatory reaction [60]. Studies suggested that extracellular HMGB1, as a late proinflammatory factor, could bind to the corresponding receptor TLRs, and activate TLR4 in this process. At present, it has been confirmed that TLR4 is a HMGB1 receptor. TLR4 can promote the expression of inflammatory factors such as TNF-a through signal transduction mechanism and mediate the immune response and tissue damage [61]. Moreover, TLR4 is combined with MYD88 through ligand proteins, and NF-kB, as their downstream important nodal protein, is a multidirectional active transcription regulatory factor, which exerts a vital effect on inflammatory response. The phosphorylation level of p65 can reflect the activation of NF-kB. Most likely, similar to oxidative stress, inflammation is usually persistent. Generally speaking, inflammation is advantageous to the body, but excessive inflammation will injure the normal tissues. Huang et al. discovered that TLR4 could aggravate lung injury by activating the MYD88/NF-κB pathway [62]. Otherwise, it could reduce intestinal inflammation and slow down intestinal barrier damages by inhibiting the MYD88/NF-κB pathway [63]. According to our results, Cd remarkably promoted the expression level of extranuclear HMGB1, and the expression levels of the HMGB1/NF-κB pathway-related factors (HMGB1, TLR4, MYD88, NF-κB and p-65/p65) were obviously elevated, while the above changes were significantly reduced after co-treatment of Se and Cd, confirming that exposure of Cd to duck hepatocytes caused the activation of the HMGB1/NF-κB pathway, which further expanded the inflammatory response. That is to say, Cd triggered oxidative stress in duck liver cells, which led to the activation of the HMGB1/NF-κ B pathway by NLRP3 inflammasome, further enlarging the inflammatory reaction. Moreover, through its antagonistic effect on Cd, Se effectively weakens Cd-induced oxidative stress and inflammatory injury.

Furthermore, inflammation and oxidative stress interact. ROS exacerbate inflammation, which in turn increases ROS production [64]. Recent studies demonstrated that HMGB1 was stimulated by inflammatory bodies of NLRB, and HMGB1 could in turn facilitate the formation of inflammatory bodies of NLRP3, forming positive feedback of inflammation, thus sustaining or even intensifying the inflammatory cascade reaction and making acute inflammatory damage [65,66]. This was also manifested in our correlation analysis. The findings revealed that there was an evident positive correlation between oxidative stress-related factors and inflammation (NLRP3 inflammasome, HMGB1/NF-κB pathway and other related inflammatory factors). Additionally, according to the results of PCA, HMGB1 is the pivotal factor affecting the oxidative stress and inflammation of liver cells induced by cadmium, which may be the key breakthrough in the treatment of Cd poisoning. Moreover, in our experiment, Se also exhibited obvious antagonism to Cd, which reduced the oxidative stress and inflammatory reaction of duck hepatocytes caused by Cd and played a certain protective role.

## 4. Materials and Methods

### 4.1. Cells Isolation and Cells Culture

We extracted cells under the approach of Yang et al. and Picardo et al. [50,67]. Briefly, the livers were taken from the 13-day-old Shaoxing duck (*Anas platyrhyncha*) embryos, then combined and chopped. The tissues were then placed in 5 mL phosphate-buffered saline (PBS) comprising 1 g/L collagenase and incubated at 37 °C for 15 min. Subsequently, dulbecco’s modified eagle medium (DMEM) containing 10% Fetal Bovine Serum (FBS) was injected into the tissues, and the cell solution was screened by nylon mesh (200 μm and 400 μm) and centrifuged twice at 200 g at 4 °C for 5 min each time. The collected precipitates were floated in the growth medium (GM) composed of dexamethasone (40 ng/mL), insulin (0.57 μg/mL), streptomycin (75 U/mL), L-glutamine (400 μg/mL), penicillin (75 U/mL) and transferrin (5 μg/mL). Thereafter, the incubated cells were confluent to 80% at 37 °C and 5% CO_2_, and then follow-up experiments were carried out.

### 4.2. Cells Viability Assay

Approximately 1 × 10^5^ primary hepatocytes per well were seeded in a 96-well culture plate and grown to 80% confluence. In accordance with the agreement of the manufacturer, the CCK-8 commercial kit (Beyotime Biotechnology, China) was adopted to monitor cell viability. The cells were exposed to variable concentration gradients of CdCl_2_ (0, 0.5, 1, 2, 4, 8, 16 and 32 μM) and Na_2_SeO_3_ (0, 0.1, 0.2, 0.4, 0.8, 1.6 and 3.2 μM) for 24 h, and then 10 μL CCK -8 solution and 100 μL fresh culture medium was used. After hatching at 37 °C for 2 h, the samples were examined by the microplate reader (ELX808, BioTek, Shoreline, WA, USA) at 450 nm.

### 4.3. Determination of LDH Release

Lactate dehydrogenase (LDH) levels in cell culture supernatants were determined by a commercially available LDH assay kit (Nanjing Jiancheng Bioengineering Institute, Nanjing, China) according to the manufacturer’s protocol, which is a measure of membrane integrity method. The culture supernatants were collected 24 h after Cd and/or Se exposure. Pyruvate standard solution and coenzyme I were injected at 37 °C for 15 min. Then, the process was terminated after the addition of 2,4-dinitrophenylhydrazine (DNPH). The absorbance per hole was estimated at 450 nm by a microplate reader.

### 4.4. Assay of Transaminase Index

Alanine aminotransferase (ALT) and aspartate aminotransferase (AST) activities of cell culture supernatants were measured utilizing commercial kits (Nanjing Jiancheng Bioengineering Institute, China) in accordance with the operating instructions. The activities of these two enzymes were tested through enzyme-linked assay, and the colorimetric product (510 nm) produced by this method was directly proportional to the pyruvic acid produced. Then, we calculated the activities by making a standard curve.

### 4.5. Measurement of Antioxidant Function Index

The activities or amount of malondialdehyde (MDA), total antioxidant capacity (T-AOC), GSH-Px and superoxide dismutase (SOD) were inspected utilizing commercial kits (Nanjing Jiancheng Bioengineering Institute, Nanjing, China), following the specification.

### 4.6. Detection of Intracellular ROS

Hepatocytes were handled with Cd and/or Se for 24 h, then digested with trypsin and gathered by centrifugation. Then, according to the manufacturer’s instructions, 2,7-Dichlorofluorescein diacetate (DCFH-DA) from Beyotime (Beijing, China) was employed as a probe to measure ROS levels in cells by flow cytometry (C6 Plus BD, Franklin Lakes, NJ, USA).

### 4.7. Immunofluorescence Staining

With reference to quondam investigation [51], the cells were cultured with rabbit anti-HMGB1 antibody (1:300) for 12 h and goat anti-rabbit antibody (1:500) for 1 h. The immunofluorescence of HMGB1 was imaged and analyzed with a confocal fluorescence microscope (TCS SP8; Leica, Wetzlar, Germany).

### 4.8. Colocalization Analysis of NLRP3 and ASC

After treating the hepatocytes with Cd and/or Se for 24 h, the adherent cells were cleaned three times using PBS and immobilized by 4% paraformaldehyde for 15 min. Then, they were blocked with HBSS containing 5% bovine serum albumin (BSA) for 30 min. The samples were hatched with the NLRP3 and ASC primary antibody (1:100) for 12 h and then incubated with the secondary antibody (1:500) for 1 h. The immunofluorescent results were scanned by a confocal fluorescence microscope (TCS SP8; Leica, Germany).

### 4.9. Preparation of Culture Supernatant

Hepatocytes were planted on 6-well plates with appropriate cell density, handled with Cd and/or Se for 24 h, and the cell culture medium was collected at termination. Then, they were centrifuged at 4000 g with ultrafiltration centrifuge tubes (4 mL/10 kd and 4 mL/3 kd, Millipore, Eching, Germany), and concentrated 4 mL cell culture medium to 200 microliters. Finally, 6× SDS-PAGE loading buffer was added and seethed (95 °C) for 10 min. The prepared specimens were stored at −80 °C for further analysis.

### 4.10. Enzyme-Linked Immunosorbent Measure (ELISA)

With reference to the approach of Wei et al. [68], after the cell broth was gathered, the concentrations of IL-18 and IL-1β were inspected by ELISA kits (Mlbio, Shanghai, China). The experiments were executed based on the instructions of the makers.

### 4.11. Real-Time Quantitative Polymerase Chain Reaction (RT-qPCR)

To measure the mRNA levels of related genes, total RNA was extracted utilizing TransZol Up (TransGen Biotech, Beijing, China). Subsequently, the specimens were applied to reverse-transcribes into cDNA by the cDNA Synthesis SuperMix (Transgen, China) and EasyScript^®^One-Step gDNA Removal for subsequent experiments. ChamQ SYBR qPCR Master Mix (Vazyme, Nanjing, China) was used to determine mRNA levels. The primers of HMGB1, MYD88, TLR4, NF-κB, IL-1β, IL-6, IL-18, TNF-α, NLRP3, ASC and caspase-1 were engineered by Primer Premier software. All reactions were performed employing a Quant Studio 7 Flex real-time PCR system (ABI 7900HT Applied Biosystems, Bedford, MA, USA). The primer sequences were listed in Table 1. GAPDH was regarded as a house-keeping gene, and the relative changes in gene mRNA levels were evaluated by the 2^−ΔΔCT^ method.

### 4.12. Western Blotting Analyses

Samples were lysed in RIPA buffer lysis containing protease inhibitors (PMSF) (Beyotime, China) at 4 °C, and used bicinchoninic acid (BCA) assay to determine the concentration. To further dilute the sample, 6× SDS-PAGE loading buffer was added and boiled (100 °C) for 10 min. An equal amount of the samples (20 μg) was acceded, electrophoresed on a 10% SDS-polyacrylamide denaturing gel, and then transferred to a polyvinylidene fluoride (PVDF) membrane. Anti-HMGB1 (1:1000; Servicebio, Wuhan, China), anti-p65 (1:1000; Bioss, Beijing, China), anti-p-p65 (1:1000; Sigma, Ronkonkoma, NY, USA), anti-TLR4 (1:1000; Sigma, USA), anti-MYD88, anti-NLRP3, anti-IL-1β, anti-pro-caspase-1 (1:500; Wanleibio, Shenyang, China), anti-ASC (1:1000; Santa Cruz Biotechnology, Santa Cruz, CA, USA), anti-GAPDH (1:5000; Bioss, China) as the primary antibody. With GAPDH as a reference, and the protein was measured and analyzed by Image J 1.48V and Image Lab 4.0 Software (Bio-Rad, Hercules, CA, USA).

### 4.13. Statistical Analysis

SPSS 26 and Microsoft Excel 2020 were implemented for statistical analysis. Statistical data analysis was executed utilizing the two-tailed student’s *t*-test or one-way analysis of variance (ANOVA) with Tukey’s post hoc test. The data were denoted as the mean ± standard deviation (SD) from at least three separate experiments, each one made in triplicate. Differences were considered notable at *p* < 0.05. Finally, we drew the test data with Image J, Image-Pro Plus and Origin 2021 software.

## 5. Conclusions

In summary, our results revealed that Cd-induced inflammatory response and oxidative stress could aggravate hepatocyte damage. The mechanism is closely related to the interaction between NLRP3 inflammasome and HMGB1/NF-κB pathway. Se could strengthen the activities of antioxidant enzymes and repress the expression levels of pro-inflammatory factors, which antagonizes the hepatotoxicity of Cd. This basic research is ongoing to better understand the potential protective mechanism of Se against Cd hepatotoxicity, which may help to identify therapeutic targets and applications in the field of Cd-dependent liver pathology. Nevertheless, this experiment is carried out in vitro, which has some limitations. In the next phase, we will verify this part in vivo experiments.

## Figures and Tables

**Table 1:** Gene primers sequence and their GenBank accession number.

Gene Name	Accession Number	Primer Sequences (5′ to 3′)
HMGB1	XM_027469875.2	Forward:AGTGTGAGGAGGCTGCGTAT Reverse: TAGACCTTTGGGGCCGTGTG
TLR4	NM_001310413.1	Forward:CACCAGTTTCACTTCCCCTTGT Reverse: GCTTTGCTAGGGATGACTCCAA
MYD88	NM_001310832.1	Forward:GCTTATAGAAAGGAGGTGTCGG Reverse: TGAAAGTCGCATTCGTCGCT
TNF-α	XM_005027491.5	Forward: GCATTTCGTTTTCCTTTTCAACT Reverse: ACCGTCTGAACTGTAACGGG
IL-1β	XM_038166869.1	Forward: TGGGCATCAAGGGCTACA Reverse: TCGGGTTGGTTGGTGATG
caspase-1	XM_038165654.1	Forward: CACTGCAAGGCACTGATTGG Reverse: CCAGGAGACGGTATCTCCAC
NLRP3	XM_005029958.4	Forward:CCAGCCTGAAGATCGGAGACCT Reverse: AGGAGCCACCCTAGAGGAGAGT
β-actin	NM_001310421.1	Forward:CAGCACGATGAAAATCAAGATCA Reverse: CAAGGGTGTGGGTGTTGGTAA
IL-6	XM_027450925.2	Forward:TGGCTTCGACGAGGAGAAATG Reverse: CGTCGTTGCCAGATGCTTTG
ASC	XM_013201308. 1	Forward: CAGCATTCTGGATCGGCTCT Reverse: ATTTTCTCCTGCCTGATGCTT
IL-18	XM_027444356.2	Forward: CCTGAAATCCCCTCCCGCTA Reverse: AGCTCATCTTCACCCTCGGT
NF-κB	XM_005017679.4	Forward:ACAACGTCCTTCATTTAGCAA Reverse: TCTGATAAAGGTCGTTCCTCA

## Data Availability

Not applicable.
